# UHF RFID Conductive Fabric Tag Design Optimization

**DOI:** 10.3390/s21165380

**Published:** 2021-08-10

**Authors:** Franck Kimetya Byondi, Youchung Chung

**Affiliations:** Information and Communication Engineering Department, Daegu University, Kyungsan City 38453, Korea; kimetyafrank@gmail.com

**Keywords:** wearable fabric RFID tag, wearable RFID tag, UHF RFID tag, RFID entrance system, conductive fabric, RFID entrance control system, wearable RFID antenna, conductive fabric RFID tag, fabric UHF tag antenna

## Abstract

This paper presents the design of a 920 MHz Ultra High Frequency (UHF) band radio frequency identification (RFID) conductive fabric tag antenna. The DC (Direct Current) resistance and impedance of the conductive fabric are measured by a DC multimeter and by a network analyzer at a UHF frequency band. The conductivities of the fabrics are calculated with their measured DC resistance and impedance values, respectively. The conductivities of the fabric are inserted into the CST simulation program to simulate the fabric tag antenna designs, and the results of the tag designs with two conductivities are compared. Two fabric UHF RFID tag antennas with a T-Matching structure, one with the name-tag size of 80 × 40 mm, and another with 40 × 23 are simulated and measured the characteristics of tag antennas. The simulated and measured results are compared by reflection coefficient S11, radar cross-section and reading range. The reading range of the 80 × 40 mm fabric tag antenna is about 4 m and 0.5 m for the 40 × 23 size tag. These fabric tags can be easily applied to an entrance control system as they can be attached to other fabrics and clothes.

## 1. Introduction

The RFID (Radio Frequency Identification) tags are generally attached to an object and used in a technology that transmits information of the object to a reader through a reader antenna [1]. Currently, the RFID system is generally applied to transportation cards [2], logistics management [3], health care [4], subway vehicle access control systems [5] etc. RFID system applications are classified according to the frequency band used [6]. The HF (High Frequency) band is 13.56 MHz and is used for short distances, and the UHF band is 840 to 960 MHz for passive tags [7]. The UHF band is 433 MHz for active tags, and the 2.45 GHz band is used for checking authentication and passport recognition. 

The exit or entrance of most buildings, such as public institutions, research institutes, broadcasting stations, etc. applies RFID technology to the entrance system or transportation card. They use the HF band RFID cards [1]. This HF tag entrance system is inconvenient, because RFID tags must be recognized in contact with the reader and recognized one by one. The use of UHF tags to access control systems instead of HF RFID tags makes it easy by allowing a sufficient recognition distance and multiple recognitions simultaneously. The reading distance need to be extended to allow convenient multiple object or people recognition.

Since the RFID tag antenna uses copper or a conductor, it is common to use a printing or etching method for fabrication. However, if a UHF RFID tag is manufactured using a conductive fabric, it is easy to attach the tag to bendable clothes, so that a person wearing the tagged cloth can be easily recognized in the RFID access control system [8,9,10].

Tag antenna manufactured using the current-flowing thread was introduced in Korea and abroad for the first time [8,9]. It was explained that there are various difficulties in sewing and embroidery machines when using conductive threads. Also, electrical fiber materials are easy to cut and can be applied inconspicuously to existing fabrics, and the use of conductive inks containing materials such as silver or copper has shown importance as the frequency increases [10,11]. Papers [12,13] had published the fabric tag for entrance control, and conducted measurements by attaching the fabric tag to the clothes and testing reading range. Measurements were performed before and after tagged clothes laundry. The results were the same. However, they used the conductance base on multimeter real resistance. This is why the measured and simulation results were not the same.

Therefore, this paper presents a design of a UHF band tag antenna using Silver Woven conductive fabric, which is easier to design and easier to manage than a tag using conductive thread. Section 2 shows the process of measuring the impedance of the fabrics for different shapes in the UHF band and shows the process of calculating the conductivity of the fabrics. Section 3 shows the process of designing the fabric UHF RFID tags and their results comparison. Section 4 concludes the work.

## 2. Resistance and Impedance Measurement by a Multimeter and a Network Analyzer

CST (Computer Simulation Technology) [14], an EM (Electro-Magnetic) simulation program, was used to design UHF RFID tags using a conductive fabric. When designing a fabric tag, it is necessary to input the conductivity value of the fabric into the simulation program. Therefore, the conductivity value of the conductive fabric needs to be measured. The impedance of the fabric depends on the shape and the thickness. To infer the conductivity value, the fabric resistance value R is measured using a multimeter [15] for low frequency fabric applications; a vector network analyzer (VNA) is used for the HF and UHF bands. The resistance measured in low frequency is lower than the one measured in higher frequencies due to a skin depth effect. At a higher frequency, the skin depth decreases, which causes the increase of the resistance [16]. We measured the impedance using VNA, then the resistivity ρ of the fabric was calculated using the resistance Formula (1) considering the thickness of the fabric, the cross-sectional area S, and the measured resistance value. The resistivity is the product of the resistance R and the cross-sectional area S, divided by length of the fabric L. The cross-sectional area, S, is the multiplication of thickness, T, and width, W in Table 1. Conductivity K is the reciprocal of the resistivity ρ Formula (2). That is, 1/ρ and has the unit of siemens per meter (S/m). Conductivity ranges from zero (for a perfect dielectric) to infinity (for a perfect conductor).

(1) $$R=\;\rho \frac{L}{S},$$

(2) $$K=\frac{1}{\rho }=\frac{R\times S}{L},$$

To find the above conductivity value, it is necessary to select a conductive fabric to be used for fabrication. The fabric adafruit-1168 is a woven conductive silver coated fabric [17]. The fabric p1364 is a knit type [18]. The fabric com-14110 is a carbon-type black fabric, and the fabric com-14111 is a compressed carbon-type fabric [19]. The resistance value increases as the length L increases in all types of fabric. The fabric com-14110 and com-14111 have a large kΩ value resistance per unit length, and p1364 and Adafruit-1168 have a relatively small Ω values resistance per unit length, so they are suitable for fabric tag design [20].

**Figure 1:**
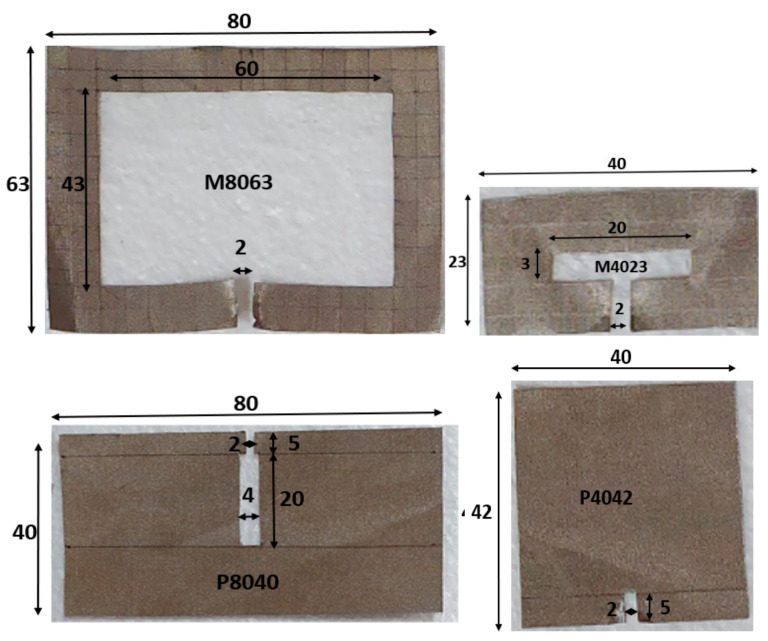
Different shapes and sizes of Adafruit-1168 fabric for impedance and resistance measurement.

**Figure 2:**
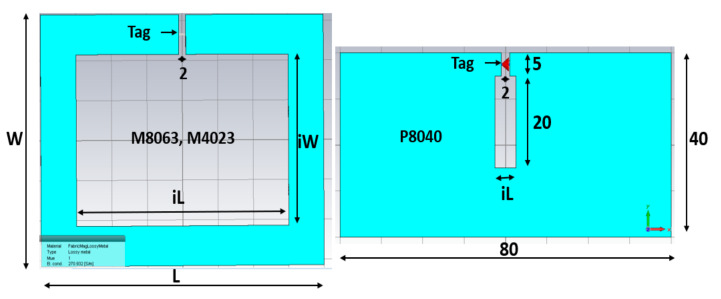
Parameters of three CFTA antenna designs (M8063, M4023, P8040).

In our paper we made different shapes and sizes using the fabric Adafruit-1168, and measure first each shape’s impedance value in UHF 920 MHz according to the size and shape. Figure 1 shows the size of the named square shape M8063 and M4023, made with conductive Adafruit-1168 fabric, and the named plain P8040 and P4042 conductive Adafruit-1168 fabric. To measure their impedance and resistance, respectively, with VNA and a digital multimeter, the probe is placed at the two opposite gap points separated by 2 mm of space. The measured impedance, resistance, and calculated conductance K are summarized in Table 1 for only four types; other types are only commented below. The current path length differs by each shape; it depends on the width and heights of the fabrics in Figure 1. The horizontal length of the current path of the fabric, Lw, can be calculated with the inner width (iW) of the fabric (outer width 80 − inner width 60 mm)/2 in Figure 1 and Table 1. Therefore, Lw for M8063 in Figure 1 is 70 mm (Lw = iW + 10). The vertical length of the current path of the fabric, Li, can be calculated with the inner vertical height (iL = 43 mm) of the fabric (outer height 63 − inner height 43 mm)/2 in Figure 1 and Table 1. Therefore, Li for M8063 in Figure 1 is 53 mm (Li = iL + 10), and iL and iW are shown in Figure 2.

For the M8063, the circumference of the current average path was 2 × (70 mm + 53 mm), which equals to 246 mm (0.246 m) in Table 1. Its resistance measured by a multimeter is 2.6 Ω, and the impedance at 920 MHz is 120 − j230 Ω measured by a VNA. For the M4023, the circumference current average path is 2 × (40 mm + 13 mm), which equals to 106 mm (0.106 m) in Table 1. Its resistance value measured by a multimeter is 2.3 Ω, and the impedance at 920 MHz is 140 + j201Ω measured by a VNA. 

P8040 and P4042 have short circumferences of the current path, as shown in Figure 1. Since the RFID strap has a 2 mm gab for bonding pads and the feeding point of the tag antenna, there is 2 mm equal gabs for 4 different shapes. For the P8040, the circumference of the current path is 2 × (25 mm + 4 mm), which equals to 58 mm. Its resistance value measured by a multimeter is 2.3Ω, and the impedance at 920 MHz is 68 + j165Ω, measured by a VNA. For P4042, it presented 2.3 Ω with a multimeter and the lowest impedance at 920 MHz 15 + j71 Ω. The P-types plain shape shows a lower impedance than square shape fabrics, because of the shorter current path than the M types.

The conductivity of the conductive fabric varies according to the size, shape and the frequency of operation. For a plain shape (P shape), the impedance is low and the conductivity is high, for the non-plain shape the impedance is high and the conductivity is low. For the low frequency, the impedance is low and conductivity is high, for the high frequency the impedance is high and the conductivity is low.

The resistivity ρ (Rho) of each shape is calculated respectively with the resistance value measured by the DC multimeter, and the impedance values measured by a VNA. The impedance has a real value of impedance (RealImp) + j imaginary value of impedance (ImagImp). The magnitude of the impedance values (MagImp) can be calculated with real and imaginary values of impedance. The conductivity is calculated using the measured resistance and impedance values by Equations (1) and (2).

The conductivity of a material should be typed into a CST EM simulation program when an antenna is simulated with any EM simulation program. The simulated reflection coefficient results of tag antenna designs have been compared based on the two different conductivities. One is the conductivity, K, of the fabric based on the DC resistance measured by a multimeter, and the other is based on the impedance measured by a VNA. This is shown in Table 1. 

## 3. UHF RFID Tag Antenna Design Using Conductive Fabric

The conductivity values of the conductive fabric tag antennas (CFTA) were entered into the CST simulation tool. The CFTA was designed to resonate at the UHF band 920 MHz frequency. The RFID chip used to make the CFTA was an Alien’s Higgs-3 chip [21]. The capacitance C value provided in the datasheet of the Higgs-3 chip is 0.85 pF, and the resistance value R is 1.5 k ohm. Therefore, the impedance value of the chip (Zc) is calculated at 920 MHz, using the Equation (3) [16], and the value is Zc = 27 − j200 Ω.

(3) $$Z_{c}=\frac{R}{1+{\;\omega }^{2}R^{2}C^{2\;}}\;\;-\;j\frac{{\omega R}^{2}C}{1+{\;\omega }^{2}R^{2}C^{2}}\;,$$

The CFTA is designed, and its impedance Za must be matched to the conjugate of chip impedance $Z_{c}^{*}$ = 27 + j200 Ω. The reflection coefficient S11 measures the quality of the match between the CFTA antenna and the chip impedances. The reflection coefficient in dB is defined by equation (4):

(4) $$S11=20\log_{10}\;\left(\frac{Z_{a}-{\;Z}_{c}^{*}}{Z_{a\;}+Z_{c}}\right),$$

The matching between source (antenna) and load (chip) can be measured through voltage standing wave ratio (VSWR) too. The reflection coefficient S11 can be determined by the measure of the standing wave caused by the superposition of the incident wave and the reflected wave. The ratio of the maximum divided by the minimum is the voltage standing wave ratio (VSWR). The VSWR is infinite for total reflections because the minimum voltage is zero. If no reflection occurs, the VSWR is 1. The VSWR and reflection coefficient are related as follows:

(5) $$\mathrm{VSWR}=\frac{1+\left|S11\right|\;}{1-\left|S11\right|\;},$$

A good match between the source (CFTA) and the load (chip) will lead to a great read-range. The read-range is an important characteristic of the tag antenna. It can be calculated using the Friis Equation (6) [22,23,24,25,26]. Where **τ** is the power transmission coefficient that determines matching between the chip and tag antenna, λ is the wavelength, EIRP is the equivalent isotropic radiated power transmitted by the reader antenna, Gr is the CFTA gain, and $P_{\mathrm{th}}$ is the minimum power required to activate the chip (from the datasheet). EIRP is the product of the readers transmitted power $P_{t}$ and reader antenna gain $G_{t}$.

(6) $$\mathrm{RR}=\frac{\lambda }{4\pi }\sqrt{\frac{P_{t}G_{t}G_{r}}{P_{\mathrm{th}}}\tau }\;0\leq \tau \leq 1,$$

The power ${\;P}_{r}$ radiated back to the reader antenna from the tag antenna can determine how big the tag antenna is, and which read distance it has. This power can be calculated using the radar cross-section (RCS) [27,28,29,30,31,32]. The RCS measures how detectable a tag antenna (object) is by reader antenna (radar). A tag antenna with a larger RCS is more easily detected. The radiated power${\;P}_{r}$ can be calculated using the radar Equation (7).

(7) $$P_{r}=K\;\frac{P_{t}G_{t}}{4{\pi R}^{2}}A_{\mathrm{eff}}G,$$

where, $K=\frac{4R_{a}^{2}}{{\left|\mathrm{Za}+\mathrm{Zc}\right|}^{2}}$ is the influence of the load (chip) impedance mismatch on the radiated power. Aeff=Gλ24π the effective area of the tag antenna, the term ${\mathrm{SA}}_{\mathrm{eff}}=\frac{P_{t}G_{t}}{4{\pi R}^{2}}A_{\mathrm{eff}}$ in the radar equation is the product of the power density S that the reader produces to the tag antenna (watts per square meter) and the effective area $A_{\mathrm{eff}}$. The product is the total power intercept by the tag antenna (in watts). G is the tag antenna gain.

Equation (7) is the backscattered power of a tag antenna for a minimum amount of power. The radar cross section (RCS) of the RFID tag can be calculated as Equation (8).

(8) $$\sigma \;=\frac{P_{r}}{S}={K\;A}_{\mathrm{eff}}\;G\;,$$

By substituting the K and $A_{\mathrm{eff}}$ data into Equation (8), the RCS become like Equation (9).

(9) $$\sigma \;=\;\frac{\lambda^{2}G^{2}R_{a}^{2}}{\pi {\left|Z_{a}+Z_{c}\right|}^{2}}\;,$$

Different conductive fabric antennas are designed for the 920 MHz frequency range, and compared by their reflected coefficient, S11, radar cross section and their reading ranges, RR.

### 3.1. Simulation of CFTA M8063, M4023 and P8040 Designs Using Different Values of K

The simulation parameter sweeps of three different CFTA is conducted to find the descent shape of antenna which will present a well-matched impedance of the antenna to the impedance of chip at 920 MHz. For the three designs, the conductivity based on UHF 920 MHz magnitude impedance (MagImp) is used. In Table 1, CFTA M8063′s magnitude of conductivity, K, is 270.9 S/m (called K271). CFTA M4023′s magnitude of conductivity, K, is 123.6 S/m (called K124). CFTA P8040′s magnitude of conductivity, K, is 185.7 S/m (called K186). These CFTA conductivities were inserted into CST, and the three designs were simulated and processed through the parameter sweep of each design. The P4042 has the conductive value of 158, where the value was located between the conductivity K = 124 and K = 186. Therefore, the parameter sweeps were only conducted for the three designs of other three designs. To have the simulation and the parameter sweeps of three antennas, the sizes, length and width of the CFTA designs of M8063, M4023 and P8040 are shown in Figure 2. 

**Figure 3:**
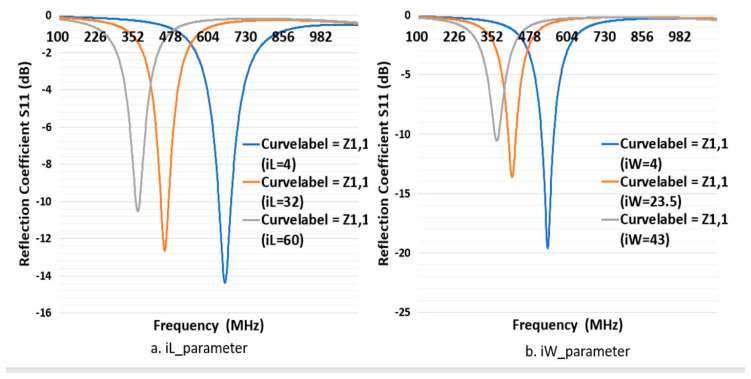
Return loss of the design CFTA M8063 by different parameters of iL and iW.

First, to simulate the CFTA M8063, the conductivity value of 270.9 S/m (K271) was inserted into CST. Its outer dimension in millimeter was L = 80 mm, W = 63 mm as shown in Figure 1. The gap in which to place the tag ship was 2 mm, and iW was 43 mm. All parameters were kept as in Figure 2, and only the parameter sweep was conducted for iL= 4, 32 and 60 mm. The parameter iL4 (4 mm) presented a simulation CFTA impedance of Za = 57 − j128Ω, iL32 (32 mm) and impedance of Za = 148 − j261 Ω. The parameter iL60 (60 mm) was the same size with the measured CFTA M8063 in Figure 1. The simulated impedance for iL60 shows Za = 100 − j242, as shown in Table 1; this value was close to the measured impedance at 920 MHz, which was Za = 120 − j230. Since the resonant frequency of iL4 was about 650 MHz, close to the 920 MHz, as shown in Figure 3, Figure 3a shows that the reflection coefficient S11 of all three parameters resonates below 700 MHz. We trace the graph to resonate at 920 MHz. The parameter iL4 resonance was closer to 920 MHz compared to other parameters. We needed to tune to reach the resonance at 920 MHz.

To sweep the CFTA M8063 by iW parameter, the outer dimensions were set as follow, L = 80, W = 63, gap 2 mm and iL was 60 mm. The parameter sweep was done for iW = 4, 23.5 mm and 43 mm. The parameter iW4 (4 mm) presented the impedance by a simulation, which was Za = 132 − j256, iW23.5 for Za = 77 − j223, and iW43 for Za = 100 − j242 at 920 MHz. Figure 3b shows that the reflection coefficient S11 of all three parameters resonates below 600 MHz. We needed to trace the graph to resonate at 920 MHz. The parameter iW4 resonate was closer to 920 MHz compared to other parameters. We needed to tune more to reach the resonance at 920 MHz. This design gives us an idea that a plain-shape and reduced size will lead to a greater result. Due to the above results, we have left this design size.

**Figure 4:**
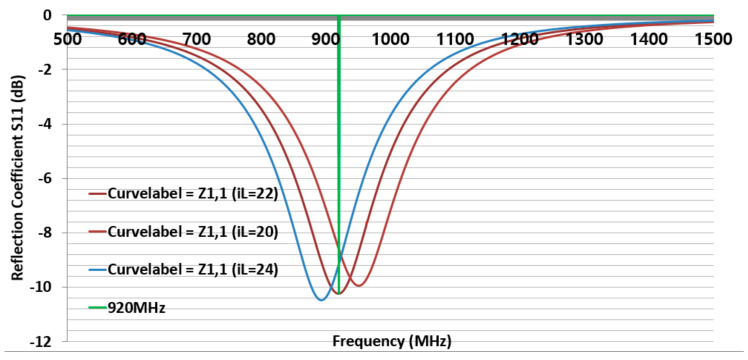
Return loss of the design CFTA M4023 by parameters of iL = 20, 22, 24 mm.

Second, to simulate the CFTA M4023, the conductivity K124 was inserted in CST. The size of M4023 was reduced compared to the size of M8063. Its outer dimensions were L = 40, W = 23, gap 2 mm, and iW was 4 mm. First, all parameters were the same as Figure 3, and only the parameter sweep was done for iL = 20, 22, and 24 mm. The parameter iL22 (22 mm) presented a simulation CFTA impedance of Za = 50 + j193 Ω. Figure 4 shows the CFTA results of the parameter sweeps, and M4023 iL = 22 mm resonated at 920 MHz. The reflection coefficient S11 was −10.2 dB. This CFTA neesded to be fabricated to measure S11 and the impedance of the antenna, Za, and to compare results.

**Figure 5:**
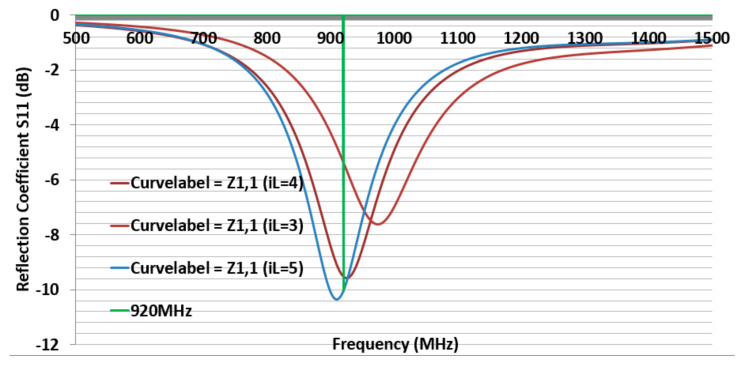
Return loss of the design CFTA P8040 by parameters of iL = 3, 4, 5 mm.

Third, to simulate the CFTA P8040, the conductivity K186 was inserted into CST. Its outer dimensions were L = 80, W = 40, gap 2 mm. First, all parameters were kept as the values shown in Figure 2; the parameter sweep was only conducted for iL =3, 4 and 5 mm. The parameter iL5 (mm) presented a simulation CFTA impedance of Za = 52 + j197 Ω. The result of parameter sweeps of iL are shown in Figure 5. Figure 5 shows that the CFTA P8040 iL5 matched with the chip at 920 MHz. The reflection coefficient S11 is −10 dB. This CFTA need to be fabricated to measure S11 and Za and to compare results. Since the last two CFTA M4023 and P8040 presented a matched result, the following study will focus on the comparison of the two antennas.

### 3.2. M4023, P8040 Comparison by Multimeter, Real, Magnitude Conductivity-K Value

The values matched for M4023 and P8040 in Section 3.1 are compared with the same design size but with different conductivity. The three conductivity K are used to simulate the M4023 and P8040 antenna designs. The conductivity K values are calculated by the DC resistance using a multimeter, the real value of the measured impedance by VNA, and the magnitude of the impedance measured by a VNA, as shown in Table 1. 

The CFTA M4023 with parameter dimensions of L = 40, W = 23, gap = 2 mm, iW is 4 mm and iL = 22 mm; P8040 with L80, W40, gap = 2, iL5, iW20 are swept by the change of conductivity-K. In Table 1, M4023 conductivity K by multimeter is 13167.7 S/m for the CFTA resistance of 2.3Ω (called K13168). K Real is 216.3 S/m (called K216) for the real impedance RealImp of 140Ω, and K Mag is 123.6 S/m (called K124) for the magnitude impedance MagImp of 244.9Ω.

For P8040, conductivity, K, by multimeter, is 14408.9 S/m for the CFTA resistance of 2.3Ω (called K14410), K Real is 487.4 S/m (called K487) for the real impedance RealImp of 68Ω, and K Mag is 185.7 S/m (called K186) for the magnitude impedance MagImp of 178.4Ω.

**Figure 6:**
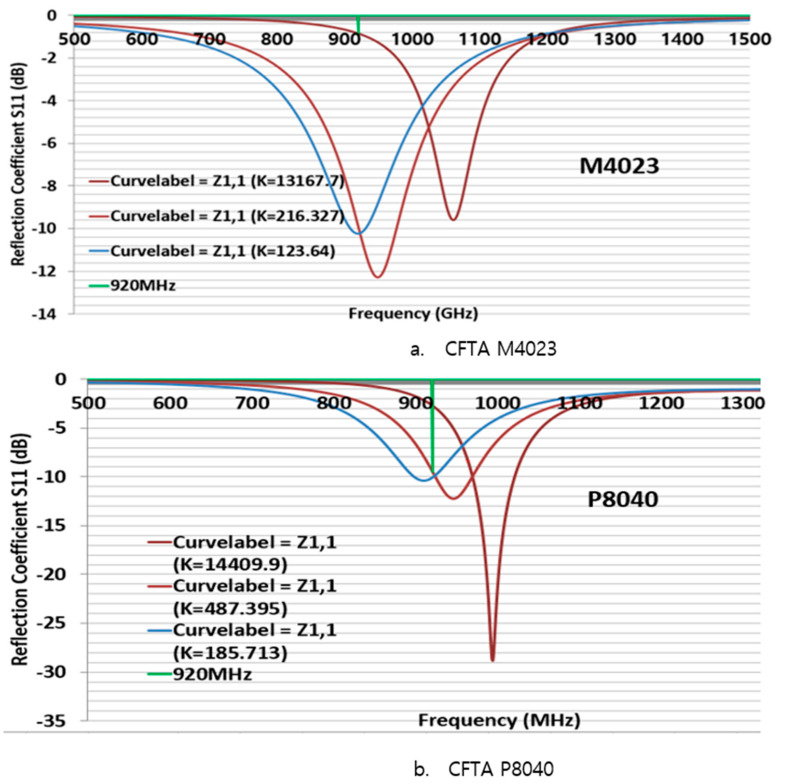
Reflection coefficient S11 comparison by conductivity-K for (**a**) CFTA M4023, (**b**) CFTA P8040.

**Figure 7:**
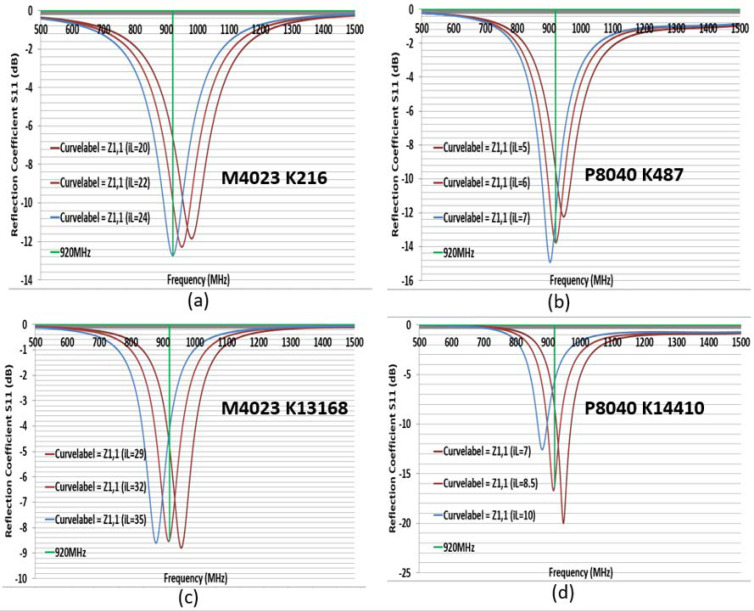
Comparison of S11 for tuned CFTA M4023 and P8040 using different K values.

Figure 6 shows the reflection coefficient S11 comparison. For a fixed size, the more the conductivity K increased, the more the resonance point moved to a higher frequency. M4023 presented K13167 impedance Za = 76 + j141 Ω; K216 Za = 39 + j182 Ω; K124 Za = 50 + j193 Ω. P8040 presented K14410 Za = 13 + j161 Ω; K487 Za = 36 + j179 Ω; K186 Za = 52 + j197 Ω. We need to tune the CFTA size for each K that it resonates at 920 MHz. The well-tuned parameter values of the CFTA M4023 K124 and CFTA P8040 K186 are, respectively, in Figure 4 and Figure 5. The well-tuned parameter values of M4023 K216, M4023 K13168, P8040 K487 and P8040 K14410 are shown in Figure 7. 

M4023 with K216 is matched with the tag for a reflection coefficient S11 = −12.7 dB, L = 40, W = 23, gap = 2, iW = 4, iL = 24 mm and the impedance Za = 43 + j196 Ω. M4023 with K13168 is matched with a tag for a reflection S11 = −8.5 dB, L = 40, W = 23, gap = 2, iW = 4, iL = 32 mm and the impedance Za = 13 + j204 Ω. P8040 with K487 is matched with the tag for a reflection coefficient S11 = −13.7 dB, L = 80, W = 40, gap = 2, iW = 20, iL = 6 mm and impedance Za = 40 + j194 Ω. P8040 with K14410 is matched with the tag for a reflection coefficient S11 = −16.3 dB, L = 80, W = 40, gap = 2, iW = 20, iL = 8.5 mm and impedance Za = 21 + j204 Ω. The matched CFTA are fabricated and their measured results are compared. 

The measured and simulation S11 results of the M4023 and P8040-based magnitude impedance conductivity showed a similar graph, both resonating at 920 MHz. Therefore, we compared only the two CFTA based magnitude impedance (MagImp) conductivities by their radar cross sections and read ranges.

### 3.3. M4023 and P8040 MagImp Simulation Radar Cross Section and Read-Range

The CFTA M4023 resonates at 920 MHz with iL = 22, magnitude impedance-based conductivity K = 124, and so it is named M4023-iL22-K124-MagImp. The CFTA P8040 resonates at 920 MHz with iL = 5, magnitude impedance-based conductivity K = 186, and so it is named P8040-iL5-K186-MagImp.

**Figure 8:**
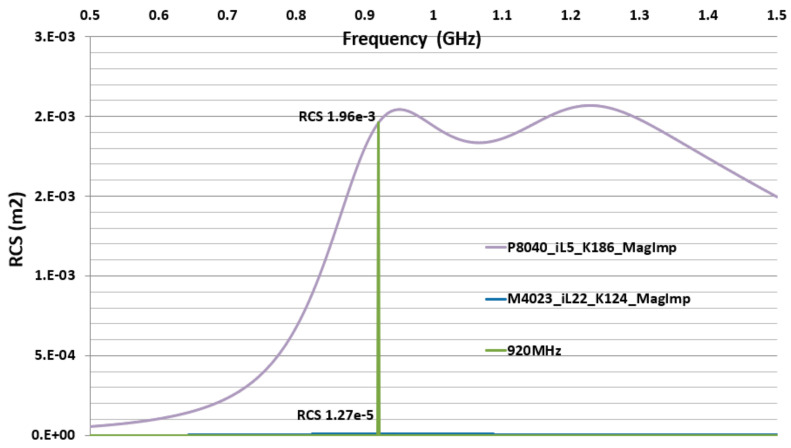
RCS comparison of CFTA M4023 and P8040.

Two CFTA M4023-iL22-K124-MagImp and P8040-iL5-K186-MagImp are compared by their radar cross-section RCS and read-ranges, RR. At the resonance frequency 920 MHz, the Higgs3 chip impedance is Zc = 27 − j200 Ω, CFTA M4023-iL22-K124-MagImp’s gain G = −15.3 dB, antenna impedance Za = 50 + j193 Ω, and antenna real impedance Ra = 50 Ω. Using Equation (9), the RCS is 1.27 × 10^−5^ $m^{2}$. CFTA P8040-iL5-K186-MagImp’s gain is G = −4.34 dB, antenna impedance Za = 52 + j197 Ω, and antenna real impedance Ra = 52 Ω. Using Equation (9), the RCS is 1.96 × 10^−3^ $\;m^{2}$. Figure 8 shows the comparison variation of the two CFTA RCS. The UHF Alien-9900 reader’s Effective Isotropic Radiation Power EIRP = $P_{t}G_{t}\;$= 4w, the minimum power to activate the Higgs3 chip, is $P_{\mathrm{th}}=-15.8\;\mathrm{dBm}$; the chip real impedance $R_{c}=27\Omega$. The matching coefficient is $\tau =\frac{4R_{a}R_{c}}{{\left|Z_{a}+Z_{c}\right|}^{2}}$. CFTA M4023-iL22-K124-MagImp’s matching coefficient τ = 0.9, and the read-range is RR = 1.64 m. CFTA P8040-iL5-K186-MagImp’s matching coefficient τ = 0.9, and the read-range is RR = 5.8 m. 

**Figure 9:**
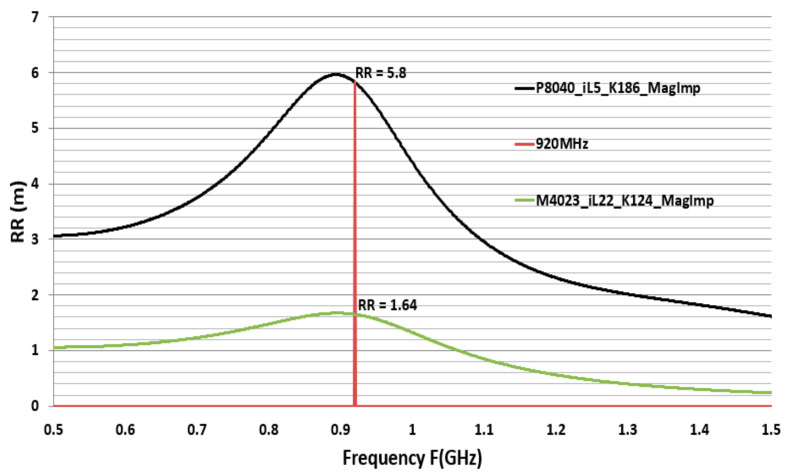
Simulation based read-range of CFTA M4023 and P8040.

CFTA P8040-iL5-K186-MagImp has a larger size, higher gain, and higher RCS than the CFTA M4023-iL22-K124-MagImp. Therefore, CFTA P8040-iL5-K186-MagImp presents a longer read-range. Figure 9 shows the variation graph of the two CFTA’s simulation based read-ranges. 

### 3.4. CFTA Fabrication—Simulation and Measurement Comparison

**Figure 10:**
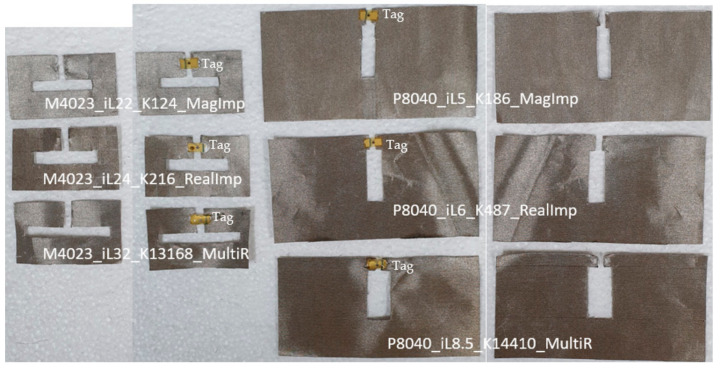
Fabricated M4023 and P8040 CFTA tag antenna optimized with different K values.

**Figure 11:**
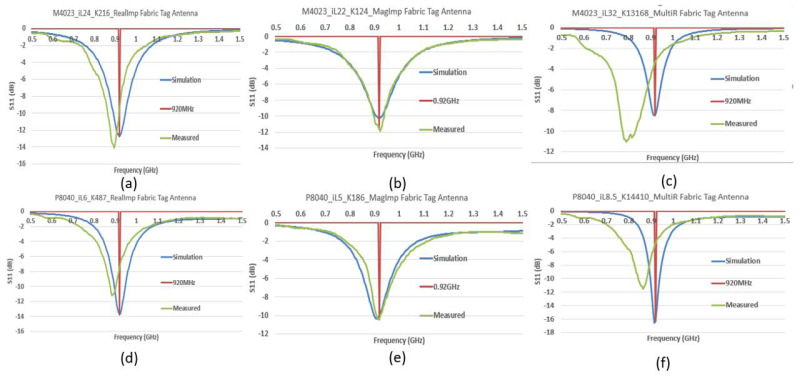
Comparison of simulated and measured reflection coefficients of M4023 and P8040.

Six different fabricated CFTA tag antennas are shown in Figure 10; CFTA without tag were used to measure the antenna characteristics such as the reflection coefficient S11 and impedance Za. CFTA with tags were used to measure the outdoor read-range. The tag was attached to the fabric antenna using conductive ELCOAT ink paste. Figure 11 compares the simulation and measured reflection coefficient S11. The CFTA designed based on conductivity K of the real impedance M4023_iL24_K216_RealImp, P8040_iL6_K487_RealImp, and conductivity based on the Multimeter resistance R M4023_iL32_K13168_MultiR, P8040_iL8.5_K14410_MultiR showed a measured reflection coefficient S11 shifted to the left. Therefore, their simulation and measured resonance frequencies differ. This is why, in the previous sections, we omitted them from the RCS and read range comparison to save time, while the CFTA designed based on the magnitude impedance M4023_iL22_K124_MagImp and P8040_iL5_K186_MagImp showed the simulation and measured reflection coefficient S11 resonance at 920 MHz. 

At 920 MHz, M4023_iL24_K216_RealImp presents a reduced measured S11 of −9.5 dB, and the simulation S11 is −12.7 dB. The difference is caused by their simulation and measured impedance results. The simulation impedance is Za = 43 + j197 Ω and the measured impedance is Za = 46 + j218 Ω. P8040_iL6_K487_RealImp presents a reduced measured S11 of −7.5 dB, and the simulation S11 is −13.7 dB. The simulation impedance is Za = 40 + j194 Ω and the measured impedance is Za = 62 + j216 Ω. M4023_iL32_K13168_MultiR presents a reduced measured S11 of −3.3 dB and the simulation S11 is −8.5 dB. The simulation impedance is Za = 13 + j204 Ω, and the measured impedance is Za = 79 + j270 Ω. P8040_iL8.5_K14410_MultiR presents a reduced measured S11 of −4.7 dB and the simulation S11 is −16.3 dB. The simulation impedance is Za = 21 + j205 Ω and the measured impedance is Za = 74 + j243. M4023_iL22_K124_MagImp presents a measured S11 of −11.7 dB and the simulation S11 is −10.2 dB. The simulation impedance is Za = 50 + j193 Ω and the measured impedance is Za = 53 + j199 Ω. P8040_iL5_K186_MagImp presents a measured S11 of −10 dB and the simulation S11 is −10.4 dB. The simulation impedance is Za = 51 + j197 Ω and the measured impedance is Za = 50 + j192 Ω. The simulation and measured impedance of the CFTA M4023_iL22_K124_MagImp are almost similar. The simulation and measured impedance of the P8040_iL5_K186_MagImp are also almost similar. Therefore, the simulation and measured reflection coefficient resonate at the same frequency of 920 MHz.

**Figure 12:**
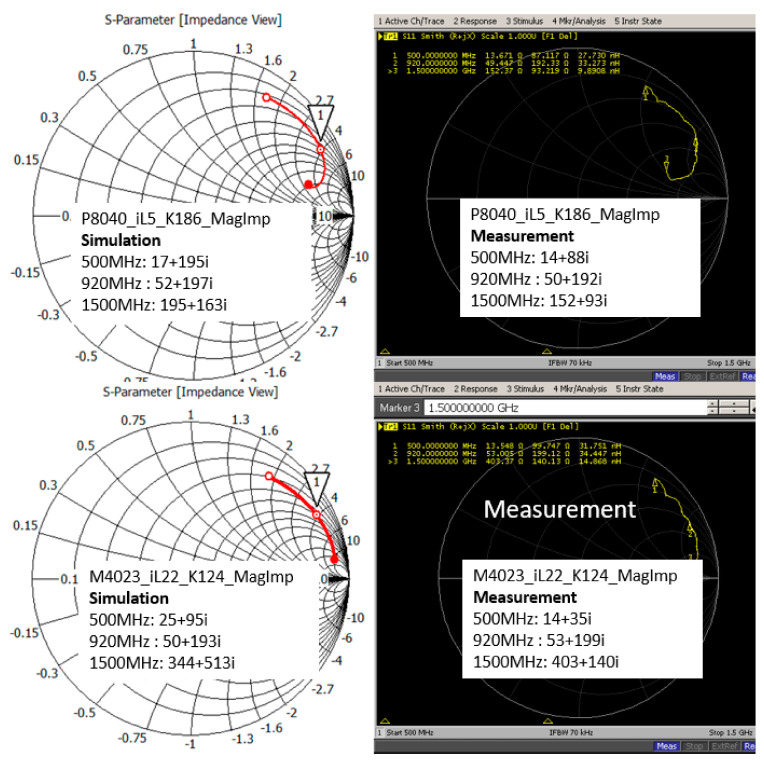
Simulation and measured impedances of the two CFTA M4023 and P8040.

Figure 12 show the simulation and measured impedances of the two CFTA. For the low frequency, the impedance is low and the conductivity is high. The frequency 500 MHz presents a lower impedance and the frequency 1500 MHz presents a higher value of the impedance. The more the frequency increases, the more the skin depth reduces, thus the resistance or impedance of the conductor (antenna) increases.

M4023 type CFTAs present a smaller radar cross section value, therefore all three M4023 types are read at the same time at a read-range of 0.25 m. The CFTA M4023_iL22_K124_MagImp has a longer read-range (0.5 m) than all CFTA M4023 types. P8040 types CFTA present a larger radar cross section value, therefore all three P8040 types are read at the same time at a read-range of 2.5 m. The CFTA P8040_iL5_K186_MagImp has a longer read-range (4 m) than all CFTA P8040 types.

The CFTA design based on the conductivity, K, calculated by the UHF magnitude impedance, presents a better performance than conductance based multimeter resistance and conductance-based UHF real impedance methods. These CFTA, especially the P8040 type, can be attached to clothes for access control into the office, hospital, schools etc. Since the P8040 types present a greater radar cross section and read-range, the advantage of the RFID UHF band is to read at a longer-range the multiple-tag antenna.

## 4. Conclusions

This paper presents the design of a 920 MHz ultra-high frequency (UHF) band radio frequency identification (RFID) conductive fabric tag antenna for access control applications. The resistance and impedance values of different shapes of the conductive Adafruit-1168 fabric are measured and calculated respectively with a multimeter and with a network analyzer at 920 MHz. The conductivities of the different shapes of fabrics are calculated using the resistance value by a multimeter, and the impedance values at 920 MHz by a network analyzer. These conductivity values are separately input into a simulation program to design the UHF CFTA tag antenna. Their simulated and fabricated measured results are compared in term of reflection coefficient, impedance, radar cross section and the read range of tag antennas. The CFTA tag antennas with a T-Matching structure and a name-tag size of 80 × 40 mm and 40 × 23 mm were simulated and fabricated. The CFTA antenna designed using the conductivity calculated with the magnitude of impedance at 920 MHz showed the better performance than other design methods that used the DC resistance value. The simulation and measurement results are well matched using the conductivity values by the impedance measured at 920 MHz. This method is to be used for designing fabric UHF tag antennas. The read-range of the best design P8040_iL5_K186_MagImp tag antenna is 4 m, the radar cross section RCS is 1.96 mm^2^, and the conductivity K is 186 S/m. The smaller CFTA M4023_iL22_K124_MagImp antenna’s read-range is 0.5 m, the RCS is 1.27 × 10^−2^ mm^2^ and the conductivity is 124 S/m. The P8040_iL5_K186_MagImp tag antenna can be applied for entrance control projects. Many people wearing the clothes embedded with this CFTA fabric tag antenna can be detected at a reasonable distance, as mentioned above.

## Figures and Tables

**Table 1:** Calculation of conductivity, K, based on the different sizes and shapes (Adafruit-1168 conductive fabric).

Size/Shape	M8063	M4023	P4042	P8040
Lw (m)	0.07	0.04	0.006	0.025
Li (m)	0.053	1.30 × 10^−2^	4.00 × 10^−3^	0.004
Thickness T (m)	0.00035	0.00035	0.00035	0.00035
Width W (m)	0.001	0.001	0.0005	0.0005
RealImp by VNA	120	140	15	68
ImagImp by VNA	−230	201	71	165
MagImp	259	245	73	179
Length = 2 × (Lw + Li) (m)	0.246	0.106	0.02	0.058
S, Area = T × W (m^2^)	0.0000035	0.0000035	1.75 × 10^−6^	0.0000175
R, by MultiMeter	2.6	2.3	2.3	2.3
Rho, by Multimeter	3.699 × 10^−5^	0.0000759	0.000201	0.0000694
Rho real by VNA	0.0017073	0.004623	0.001313	0.002052
Rho Mag by VNA	0.003691	0.008088	0.00635	0.005385
K Real at 920 MHz by VNA	586.7	216.3	762.9	487.4
K Mag at 920 MHz by VNA	270.9 (K271)	123.6 (K124)	158.5	185.7 (K186)
K by Multimeter	27033	13167.7	4968.9	14409.9

## Data Availability

Data sharing not applicable.
